# Single-nucleus transcriptome analysis reveals transcriptional profiles of circadian clock and pain related genes in human and mouse trigeminal ganglion

**DOI:** 10.3389/fnins.2023.1176654

**Published:** 2023-05-11

**Authors:** Yanhao Chu, Yaqi Wu, Shilin Jia, Ke Xu, Jinyue Liu, Lijia Mai, Wenguo Fan, Fang Huang

**Affiliations:** ^1^Hospital of Stomatology, Sun Yat-sen University, Guangzhou, China; ^2^Guangdong Provincial Key Laboratory of Stomatology, Guangzhou, China; ^3^Guanghua School of Stomatology, Sun Yat-sen University, Guangzhou, China

**Keywords:** single nucleus RNA sequencing, trigeminal ganglion, circadian clock, pain rhythms, clock gene, chronic pain

## Abstract

**Introduction:**

Clinical studies have revealed the existence of circadian rhythms in pain intensity and treatment response for chronic pain, including orofacial pain. The circadian clock genes in the peripheral ganglia are involved in pain information transmission by modulating the synthesis of pain mediators. However, the expression and distribution of clock genes and pain-related genes in different cell types within the trigeminal ganglion, the primary station of orofacial sensory transmission, are not yet fully understood.

**Methods:**

In this study, data from the normal trigeminal ganglion in the Gene Expression Omnibus (GEO) database were used to identify cell types and neuron subtypes within the human and mouse trigeminal ganglion by single nucleus RNA sequencing analysis. In the subsequent analyses, the distribution of the core clock genes, pain-related genes, and melatonin and opioid-related genes was assessed in various cell clusters and neuron subtypes within the human and mouse trigeminal ganglion. Furthermore, the statistical analysis was used to compare the differences in the expression of pain-related genes in the neuron subtypes of trigeminal ganglion.

**Results:**

The present study provides comprehensive transcriptional profiles of core clock genes, pain-related genes, melatonin-related genes, and opioid-related genes in different cell types and neuron subtypes within the mouse and human trigeminal ganglion. A comparative analysis of the distribution and expression of the aforementioned genes was conducted between human and mouse trigeminal ganglion to investigate species differences.

**Discussion:**

Overall, the results of this study serve as a primary and valuable resource for exploring the molecular mechanisms underlying oral facial pain and pain rhythms.

## Introduction

Chronic pain, which is a persistent or recurrent pain state lasting for more than 3 months (Treede et al., 2015), is a major global health concern affecting approximately 20% of the world’s population. Among various pain conditions, chronic orofacial pain (COFP) is a prevalent pain condition that affects the head, face, neck, and all intraoral structures (Araújo-Filho et al., 2018). Orofacial pain can be caused by a variety of factors, such as dental problems, temporomandibular disorders (TMD), infections, and injuries, and can also be associated with certain medical conditions, such as cancer, nerve damage, and psychiatric disorders. COFP has a prevalence of 7%–11% in the general population and 50% in the elderly population.

Animals exhibit circadian rhythms in many physiological and behavioral processes, including temperature fluctuations, sleep, metabolism, endocrine homeostasis, and immunity (Buhr et al., 2010; Huang et al., 2011; Scheiermann et al., 2013; Gnocchi and Bruscalupi, 2017). To adapt to the daily environmental light/dark cycles, animals have evolved internal timing systems known as circadian clocks. The circadian clocks are endogenous and cell-autonomous oscillators that are synchronized with the 24-h solar day (Albrecht, 2012; Takahashi, 2017), which enables animals to coordinate their physiological functions with the external environment by regulating gene expression and protein concentration. The core molecular mechanism of circadian clocks is the transcription–translation feedback loops (TTFLs) involving multiple clock genes including *CLOCK*, *BMAL1*, *PER*, *CRY*, *ROR*, and *REV-ERB*, etc. (Partch et al., 2014; Chu et al., 2022).

Pain-related diseases and treatments also display circadian rhythms. Several chronic pain conditions, such as osteoarthritis, rheumatoid arthritis, fibromyalgia, and biliary pain, show diurnal fluctuations in pain intensity, as observed in clinical studies (Harkness et al., 1982; Minoli et al., 1991; Bellamy et al., 2002, 2004; Caumo et al., 2019). Recently, circadian rhythms of pain have become a topic of interest. Clinical evidence indicates that the pain intensity of orofacial pain, including cluster headache, migraine, temporomandibular disorder (TMD), and burning mouth syndrome, also exhibits circadian rhythms (Glaros et al., 2008; Lopez-Jornet et al., 2015; Burish et al., 2018, 2019; de Coo et al., 2019; Poulsen et al., 2021). BMAL1:CLOCK has been shown to modulate circadian inflammatory pain by regulating the expression of substance P (SP) in the dorsal root ganglion (DRG) (Zhang et al., 2012).

The trigeminal ganglion (TG) serves as a collection of primary sensory neuronal cell bodies, and receives sensory information projections, including touch, pain, and temperature, from the orofacial regions. Several pain-related mediators such as neuropeptides, nitric oxide, cytokines, adenosine triphosphate, and neurotrophic factors are located in the TG. Their expression levels and related receptors affect the transmission of maxillofacial nociception (Messlinger and Russo, 2019). These pain-related mediators also exhibit circadian rhythms in their expression levels (Chu et al., 2022). Evidence suggests that clock genes are involved in the circadian rhythms of pain by influencing the rhythmic synthesis of pain-related mediators (Zhang et al., 2012; Morioka et al., 2016). However, there is a limited understanding of the role of sensory ganglia in modulating pain rhythms, particularly the role of TG in the rhythms of orofacial pain. Moreover, there is little knowledge regarding the expression and localization of circadian clock genes and pain-related mediators in different cell types of TG, including primary sensory neurons, satellite glial cells (SGCs), Schwann cells (SCs), etc.

Single nucleus RNA sequencing (snRNA-seq) or single cell RNA sequencing (scRNA-seq) is an effective and relatively new tools for generating high-resolution gene expression profiles of tissues or organs at the single-cell level. In neurobiology, snRNA-seq has shown to be a valuable technique for identifying novel cell subtypes and characterizing different cell types and states within tissues (Van Hove et al., 2019; Tran et al., 2021) compared to traditional methods.

Therefore, the aim of this study was to create a comprehensive cell expression atlas of circadian clock and pain-related genes in human and mouse TG under physiological conditions, using snRNA-seq data. By identifying gene expression patterns in cell clusters and neuron subtypes within the TG, this atlas provides a valuable resource for investigating the molecular mechanisms of orofacial pain rhythms.

## Materials and methods

### Data acquisition and processing of gene expression matrix

This study used snRNA-seq data from both mouse and human TG, which were obtained from the Gene Expression Omnibus (GEO) with the accession number GSE 197289 (Yang et al., 2022). Data from the human and mouse normal trigeminal ganglia without neurological disorders were included in the follow-up analysis of this study.

According to the protocol, 10x Cell Ranger software (version 2.0.1; https://www.10xgenomics.com) was used to process the raw sequencing data, including genome alignment, filtering, barcode and unique molecular identifier (UMI) counting by the “cellranger-count” function. Subsequently, gene-barcode expression matrices of snRNA-seq were generated for both the human and mouse TG.

For downstream quality control (QC), normalization, visualization, clustering analysis and gene expression analysis were performed with the Seurat R package (version 4.0.2) and R software. During quality control analysis, raw matrix data were filtered with standard quality control metrics: including the detection of genes in each cell (nFeature_RNA > 400); total UMIs < 15,000, percentage of reads about the mitochondrial genome in the single transcriptome of the TG (<5%); and percentage of reads mapping to ribosomal proteins (<10%). The filtered count matrices were normalized by the total gene expression and multiplied by a scale factor of 10,000 followed by log transformation using Seurat’s NormalizeData function.

### Clustering of human and mouse TG data

After the filtering and normalization procedure, the top 3,000 variable genes were identified by the FindVariableFeatures function in Seurat, which served as the basis for the subsequent clustering analysis. Principal component analysis (PCA) was conducted to identify the top 50 highly variable features/genes by the ElbowPlot function in Seurat. Unsupervised hierarchical clustering based on principal components of the most variably expressed genes was performed using the FindClusters function based on Shared Nearest Neighbor (SNN) graph and SNN modularity optimization at an optimized stable resolution. The cell clusters of the human and mouse TG were visualized using the Uniform Manifold Approximation and Projection (UMAP) method, respectively. In addition, these different clusters were identified by analyzing the expression of canonical cell markers. The cell cluster markers were identified using differential gene expression analysis, which identified genes expressed by each cell cluster when compared to all other clusters, and the percentage of cell clusters expressing these marker genes exceeded 25%, using the FindAllMarkers function of Seurat. The non-parametric Wilcoxon rank sum test was performed in the differential gene analysis, with the *p*-value set at <0.01. The calculated top marker genes of the cell clusters were compared with other published scRNA-seq studies of the nervous system (Avraham et al., 2020; Yang et al., 2022; Chu et al., 2023) to determine the identity of the cell clusters including neurons, glial cells, and other non-neural cells in the TG. The annotations of the cell clusters in the TG were validated by automated cell annotation using the SingleR R package v1.4.1. The top 10 marker genes for each cell cluster were displayed using gene heat maps.

### Subclustering of neurons data

After annotating the cell clusters in the human and mouse TG data, the neurons of human and mouse TG were extracted for subsequent subclustering analysis. The GetAssayData function in Seurat was used to extract the data of the target cell clusters from the overall TG data, and the resulting matrix file was subjected to quality control and normalization procedures as described earlier. To identify new cell subpopulations within the neurons, further unsupervised clustering analysis was performed based on the first 20 principal components, which were determined using the ElbowPlot function. The subclusters of the target cell types were visualized using UMAP analysis. The marker genes for these cell subpopulations were presented in violin plots and UMAP plots.

### Cell expression atlas of rhythm and pain-related genes in TG

Cell type expression patterns of biological clocks or rhythm-related genes, and pain-related genes in the normal TG of humans and mice were analyzed based on snRNA-seq data. The gene set of the core molecular clock of circadian clocks was obtained from a literature search and the Pubmed database. Annotated gene sets associated with biological process (BP) of Gene Otology terms, including entrainment of circadian clock, response to pain, and detection of stimulus involved in sensory perception of pain, were also used. Additionally, a final set of genes related to the regulation of opioid peptide action and melatonin action targets was obtained from a comprehensive literature search (Liu et al., 2019). After obtaining the gene sets, the expression and distribution of these gene sets were analyzed in the primary cell clusters of the human and mouse TG, respectively, as well as in subpopulations of neurons. Genes expressed in fewer than 5% of the cell clusters were not displayed in dot plots.

### Statistical analysis

Seurat’s FindMarkers or FindAllMarkers features were used for statistical analysis in the processing of snRNA-seq data for analysis of TG. The significance of differential gene expression was assessed using the Wilcoxon Rank Sum test, with a significance level of *p* < 0.01.

## Results

### snRNA-seq reveals the cell types in TG

**Figure 1:**
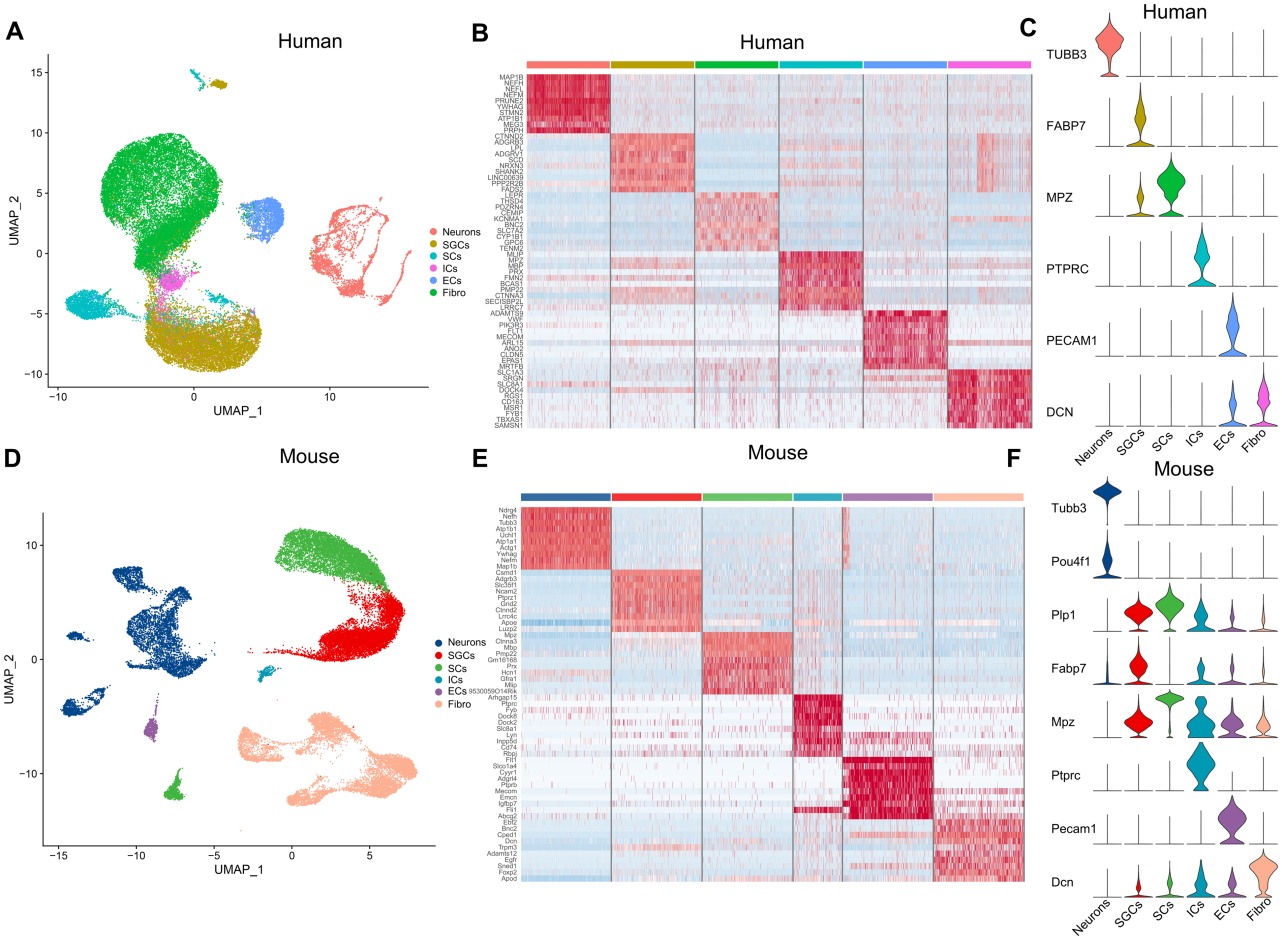
The cell atlas of human and mouse TG by snRNA-seq. **(A)** The UMAP plot demonstrates clustering of the human TG including Neurons, SGCs (satellite glial cells), SCs (Schwann cells), ICs (immune cells), ECs (endothelial cells), and Fibro (Fibroblasts). Each dot represents a cell; each color represents a cluster or a cell type. **(B)** The heat map of the top 10 genes for the cell clusters of the human TG. The row represents cell type, indicated by different colors. The column represents the top 10 markers for each cell type. **(C)** The violin plot indicates the signature marker genes of the cell clusters of the human TG. **(D)** The UMAP plot demonstrates clustering of mouse TG. **(E)** The heat map of the top 10 genes for the cell clusters of the mouse TG. **(F)** The violin plot indicates the signature marker genes of the cell clusters of the mouse TG.

The snRNA-seq data of the human and mouse TG, which were obtained from the dataset GSE 197289 in the GEO database, were analyzed in this study. Following quality control procedures, a total of 37,973 single nuclei were detected in the human TG data, and 28,808 nuclei in the mouse TG. We performed data-driven clustering to generate cell clusters in the TG, which showed similar cell types for the human and mouse TG. To identify marker genes of cell clusters, we calculated the expression difference of each gene between that cluster and the average in the rest of the clusters (ANOVA fold change threshold > 1.5). The marker genes for the cell clusters in this study were identified by further reference to published snRNA-seq or scRNA-seq studies on the DRG and TG (Hillmer et al., 2016; Avraham et al., 2020; Yang et al., 2022). The cell clusters identified in the human and mouse snRNA-seq data include the following six main categories: neurons, satellite glial cells (SGCs), endothelial cells (ECs), immune cells (IMs) (mainly macrophages or macrophage precursor cells), Schwann cells (SCs), and fibroblasts (Figures 1A,D). Using differential expression analysis, the top 10 preferentially expressed genes of the main cell clusters in the human and mouse TG were shown in Figures 1B,E, respectively. In our analysis, the signature genes of the cell clusters were as follows: neurons (*TUBB3*), SGCs (*FABP7*), SCs (*MPZ*), ICs (*PTPRC*), ECs (*PECAM1*), and fibroblasts (*DCN*) in human TG (Figure 1C). The mouse TG has similar cell clusters and cell markers: neurons (*Tubb3*, *Pou4f1*), SGCs (*Plp1*, *Fabp7*), SCs (*Mpz*), ICs (*Ptprc*), ECs (*Pecam1*), and fibroblasts (*Dcn*) (Figure 1F).

### The expression profile of core circadian clock genes in TG

**Figure 2:**
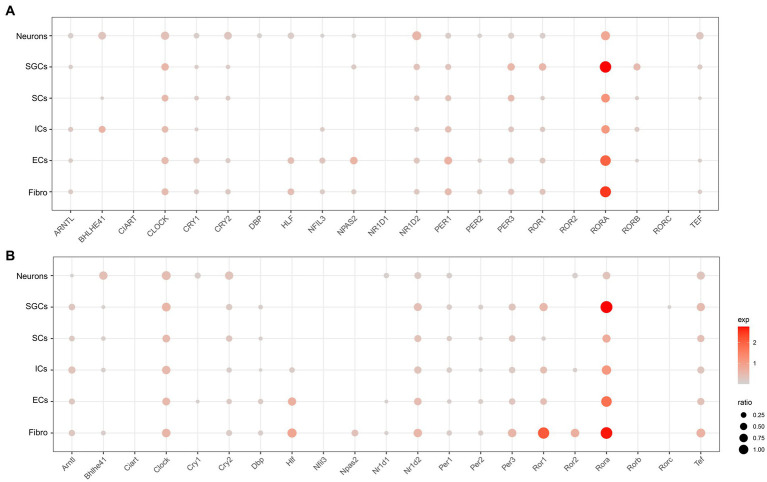
Transcriptional profiles of core circadian clock genes in the TG cell clusters. **(A)** The dot plot showing the expression of core circadian clock genes in the human TG cell clusters. The dot size is proportional to the percentage of each cluster expressing the gene (pos_pct), and the color intensity is correlated with the expression level (avg_exp). Dots with pos_pct < 5% are not shown **(B)**. The dot plot showing the expression of core circadian clock genes in the mouse TG cell clusters.

To understand the cell type-specific expression of clock genes for the human and mouse TG, we obtained a list of genes about the core clock genes and then used dot plots to show the cellular transcriptional landscape of the core clock genes in human (Figure 2A) and mouse TG (Figure 2B) by snRNA-seq, respectively. The core biological clock is composed of a conserved group of genes and the corresponding encoded proteins, which form the transcriptional-translational feedback loop (Patke et al., 2020). The core molecular clock mainly consists of *BMAL1* (Brain and Muscle ARNT-like 1) or *ARNTL*, *CLOCK* (Circadian Locomotor Output Cycles Kaput), *PER1/2* (Period 1/2), *CRY1/2* (Cryptochrome 1/2), *ROR* (RAR Related Orphan Receptor), etc. In the molecular clock, *RORA* (Retinoic Acid Receptor-Related Orphan Receptor Alpha) was richly expressed in various cell clusters in both the human (Figure 2A) and mouse TG (Figure 2B), particularly in SGCs and fibroblasts for both the human and mouse TG (>70% of the cell population), with a higher expression percentage in human neurons (46%) compared to mouse neurons (28%) (Figures 2A,B). *ARNTL*, *CLOCK*, *CRY2*, *NR1D2*, *PER1*, *PER3*, and *TEF* show consistent expression patterns in both the human (Figure 2A) and mouse (Figure 2B) TG, i.e., evenly positive expression in different cell clusters. *CRY1*, a negative feedback regulator of the molecular clock that inhibits ARNTL/CLOCK heterodimers (Albrecht, 2012), is expressed in a higher proportion in the human TG than in the mouse TG. *Cry1* expression was only slightly higher in mouse neurons (ratio = 15.6%) compared to other cell populations of the mouse. The expression ratio of *PER2* in the human TG is higher than 5% only in neurons, fibroblasts, and endothelial cells (Figure 2A), compared to the cell types in the mouse TG (Figure 2B). Furthermore, *NR1D1*, which encodes the REV-ERBα protein and negatively regulates the expression of core clock components *ARTNL/BMAL1*, was expressed at very low proportions (<5%) in various cell clusters in the human TG, and at a higher proportion (11.3%) in mouse neurons.

### The expression profile of pain-related genes in TG

**Figure 3:**
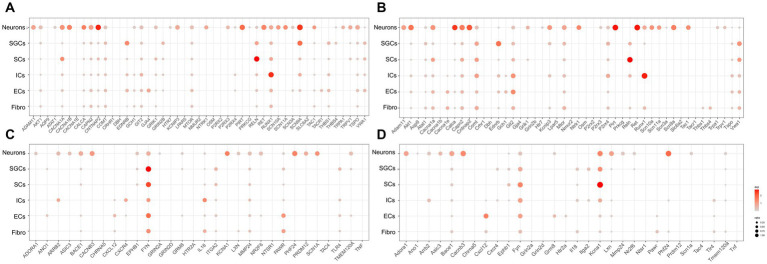
Transcriptional profiles of pain-related genes in the TG cell clusters. **(A)** The dot plot showing the expression of genes associated with “response to pain” in the human TG cell clusters. **(B)** The dot plot showing the expression of genes associated with “detection of stimulus involved in sensory perception of pain” in the human TG cell clusters. **(C)** The dot plot showing the expression of genes associated with “response to pain” in mouse TG cell clusters. **(D)** The dot plot showing the expression of genes associated with “detection of stimulus involved in sensory perception of pain” in the human TG cell clusters.

To investigate the localization of pain-related genes in the cell clusters of TG, we obtained sets of pain-related genes and then used dot plots to show their distribution in the TG. The resultant analysis mainly focused on the distribution of pain-related genes in neurons, SGCs, SCs, and ICs. The genes associated with “response to pain” in the human and mouse TG were shown in Figures 3A,B, respectively. Our findings demonstrate that several genes are expressed more broadly and specifically in human TG neurons: *ADAM11* encoding the ADAM (a disintegrin and metalloprotease) protein, *CACNA1B* encoding the N-type voltage-dependent calcium channel pore-forming subunit, *CALCA* encoding calcitonin related polypeptide alpha, *CAPN2* encoding calcium-activated neutral proteases, *CNTNAP2* encoding a neurexin family member, KCNIP3 encoding potassium voltage-gated channel Interacting protein 3, *NTRK1* encoding the tyrosine kinase receptor, *P2RX3* encoding the purinergic receptor P2X3, *PIRT* encoding the phosphoinositide interacting regulator of transient receptor potential channels, *RET* encoding proto-oncogene C-ret, and genes associated with voltage-gated sodium channels (*SCN11a*, *SCN10a*, *SCN3a*, and *SCN9a*), as well as the transient receptor potential cation channel-related genes *TRPA1* and *TRPV1* (Figure 3A). In contrast, neurons in mouse TG have similar expression and localization of the above genes, but the proportion of *Cacna1b* expression is lower in mouse neurons (percent < 1% for mouse neurons and percent = 55.8% for human). As shown in Figures 2A,B, *EDNRB* (Endothelin Receptor Type B), which encodes the G protein-coupled receptor, was enriched in SGCs in both human and mouse TG. Studies have shown that upregulation of EDNRB in astrocytes promotes the activation of astrocytes in allergic and atopic disorders (Asada et al., 2004; Yamasaki et al., 2016). *GRIN2B* (Glutamate Ionotropic Receptor NMDA Type Subunit 2B), which encodes a subunit of the NMDA receptor, namely NR2B, has been suggested to be elevated in SGCs of DRG in a spared nerve injury (SNI) model (Hogan-Cann and Anderson, 2016; Norcini et al., 2016). Our results indicate that SGCs in human TG express higher levels of *GRIN2B* under physiological conditions, whereas mouse SGCs express very low levels. Lastly, compared to other cell populations, *RUNX1* (RUNX Family Transcription Factor 1) has a greater proportion of expression in immune cells in both human and mouse TG (Figures 2A,B).

The genes associated with “detection of stimulus involved in sensory perception of pain” in the cell clusters in human and mouse TG were shown in Figures 3C,D, respectively. The results indicated that *ADORA1* (Adenosine A1 Receptor), *ARRB2* (Arrestin Beta 2), *ASIC3* (Acid Sensing Ion Channel Subunit 3), *BACE1* (Beta-Secretase 1), *CACNB3* (Calcium Voltage-Gated Channel Auxiliary Subunit Beta 3), *LXN* (Latexin), *NR2F6* (Nuclear Receptor Subfamily 2 Group F Member 6), *PHF24* (PHD Finger Protein 24), *PRDM12* (PR/SET Domain 12), *SCN1A* (Sodium Voltage-Gated Channel Alpha Subunit 1) were specifically highly expressed in neurons relative to the other cell clusters in both human and mouse TG. In addition, *FYN* (FYN Proto-Oncogene), which encodes a membrane-associated tyrosine kinase and is important for myelination and synaptic transmission in the central neural system (CNS) (Chun et al., 2004; Guglietti et al., 2021), was demonstrated to be enriched in SGCs and SCs in both human and mouse TG (Figures 3C,D). *MMP24* (Matrix Metallopeptidase 24), which increased gradually in the spinal cord after the sciatic nerves were partially ligated (Liou et al., 2013), was expressed at a higher proportion in human SGCs (percent = 12.5%) than in mouse SGCs (percent < 1%) (Figures 3C,D). *KCNA1* (Potassium Voltage-Gated Channel Subfamily A Member 1) encodes a voltage-gated delayed potassium channel known as Kv1.1, and is predominantly expressed in neurons, SGCs, and Schwann precursor cells (Hallows and Tempel, 1998). Kcna1-null mouse also exhibited pain hypersensitivity (Jiang et al., 2003). Our data showed that in mouse TG, *Kcna1* was expressed in many cell clusters, with higher proportions in neurons (percent = 51.1%), SGCs (percent = 47.9%), and SCs (percent = 84.0%) (Figure 3D). However, in the human TG, *KCNA1* expression was observed in neurons (percent = 49.1%) and to a much lesser extent in SGCs (percent = 1.7%) and SCs (percent = 3.1%), compared to its expression in mouse TG (Figure 3C). *TLR4* (Toll-Like Receptor 4), which plays a role in pathogen recognition and innate immunity, is associated with pain (Navia-Pelaez et al., 2022) and has been validated for expression in SGCs of human and rat TG (Tse et al., 2014; Mitterreiter et al., 2017). The results of this study further verified that *Tlr4* was significantly expressed in the SGCs of the human TG (20%) (Figure 3C), but was poorly expressed in mouse SGCs (1%) (Figure 3D). Moreover, as shown in Figures 3C,D, *ARRB2* (Arrestin Beta 2), *CXCR4* (C-X-C Motif Chemokine Receptor 4), and *IL18* (Interleukin 18) were clearly expressed in both human and mouse immune cells.

In another published study, a search aggregated a list of pain genes obtained from a series of pain databases as well as relevant literature sources for pain-related genes as well as their family genes (Fang et al., 2022). We also analyzed the distribution of genes from the above pain gene list in cell clusters of the human and mouse TG and selected the output of genes with expression percentages greater than 5%, as shown in Supplementary Tables S1, S2.

### The expression profile of melatonin and opioid-related genes in TG

**Figure 4:**
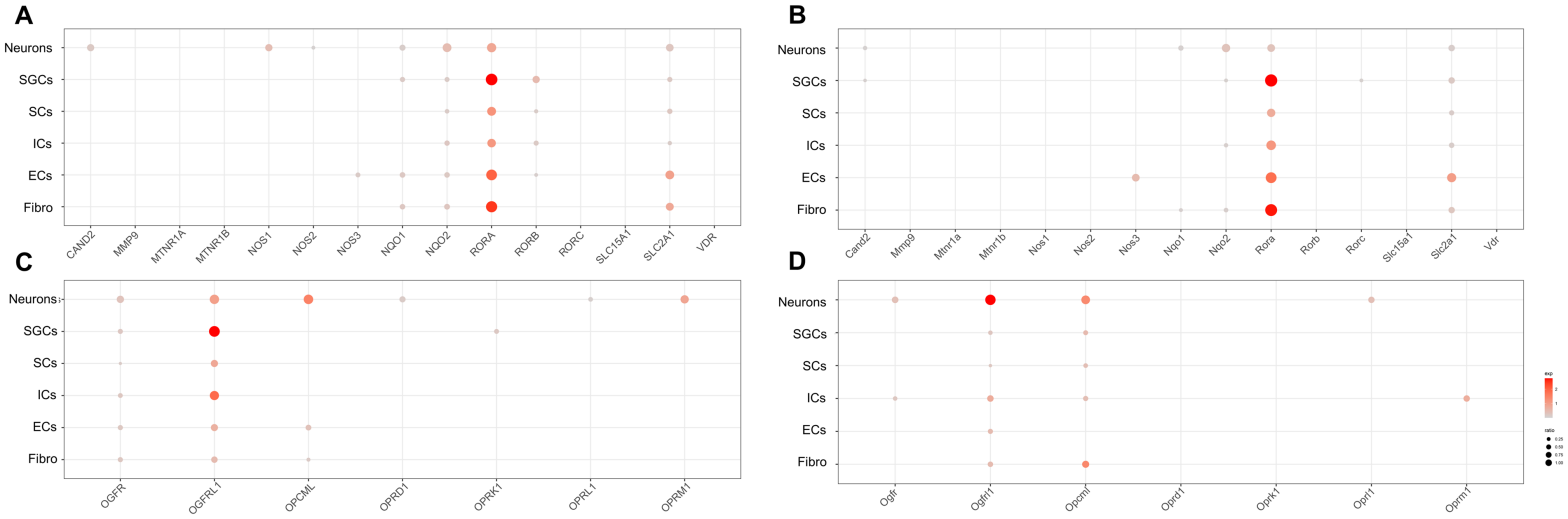
Transcriptional profiles of melatonin and opioid-related genes in the TG cell clusters. **(A)** The dot plot showing the expression of melatonin target genes in the human TG. **(B)** The dot plot showing the expression of melatonin target genes in the mouse TG. **(C)** The dot plot showing the expression of opioid-related genes in the human TG. **(D)** The dot plot showing the expression of opioid-related genes in the mouse TG.

Melatonin, the hormone secreted by the pineal gland, plays a crucial role in regulating the sleep–wake cycle and other circadian rhythms in physiological functions (Cajochen et al., 2003; Vasey et al., 2021). Many studies have demonstrated that melatonin can provide alleviative and neuroprotective effects for chronic pain (Kaur and Shyu, 2018). Opioids, including endogenous opioids like beta-endorphins and exogenous opioids such as morphine, are closely associated with the circadian rhythm of bodily functions (Reid et al., 1982). The analgesic effects of morphine and its addiction are also linked to changes in the expression of clock genes (Roy et al., 2021). Therefore, in order to investigate the distribution of possible melatonin target proteins (Liu et al., 2019) and opioid-related genes, including various opioid receptor genes, in the peripheral nervous system, we obtained possible melatonin target genes and opioid-related genes and used dot plots to demonstrate their expression in various cell clusters of the human (Figure 4A) and mouse (Figure 4B) TG.

Regarding melatonin-related genes, our data showed that *CAND2* (Cullin Associated and Neddylation Dissociated 2), *NQO1/2* (NAD(P)H Quinone Dehydrogenase 1/2), and *SLC12A1* (Solute Carrier Family 12 Member 1) were significantly expressed in neurons of the human and mouse TG (Figures 4A,B). Notably, *NOS1* expression in neurons was higher in human TG (23.4%) than in mouse TG (<2%). Additionally, *RORB* (RAR Related Orphan Receptor B) and *NQO1* were prominently expressed in SGCs of the human TG (Figure 4A), but were difficult to detect in mouse SGCs (Figure 4B). Moreover, about 9.2% of human immune cells expressed *RORB* (Figure 4A), whereas mouse ICs exhibited almost no *RORB* expression (Figure 4B).

The expression of opioid-related genes in the human and mouse TG was illustrated in Figures 4C,D, respectively. In the human TG neurons, a rich expression of opioid-related genes was observed, including *OGFR* (Opioid Growth Factor Receptor), *OGFRL1* (Opioid Growth Factor Receptor Like 1), and *OPCML* (Opioid Binding Protein/Cell Adhesion Molecule Like), as well as opioid receptor-associated genes including *OPRD1* (Opioid Receptor Delta 1), *OPRL1* (Opioid Related Nociceptin Receptor 1), and *OPRM1* (Opioid Receptor Mu 1) (Figure 4C). In contrast, a smaller number of opioid-related genes were apparently expressed in neurons of the mouse TG, including *Ogfr*, *Ogfrl1*, *Opcml*, and *Oprl1*. Notably, *Oprm1* was specially expressed in immune cells of mouse TG (percent = 16.7%), whereas its expression was comparatively lower in immune cells of the human TG (3.0%) (Figure 4D). As for SCs of TG, *OGFR* and *OGFRL1* were clearly detectable in human SCs (percent > 5%) (Figure 4C), while *Ogfrl1* and *Opcml* were clearly detectable in mouse SCs (percent > 5%) (Figure 4D).

### snRNA-seq reveals the neuron subtypes in TG

**Figure 5:**
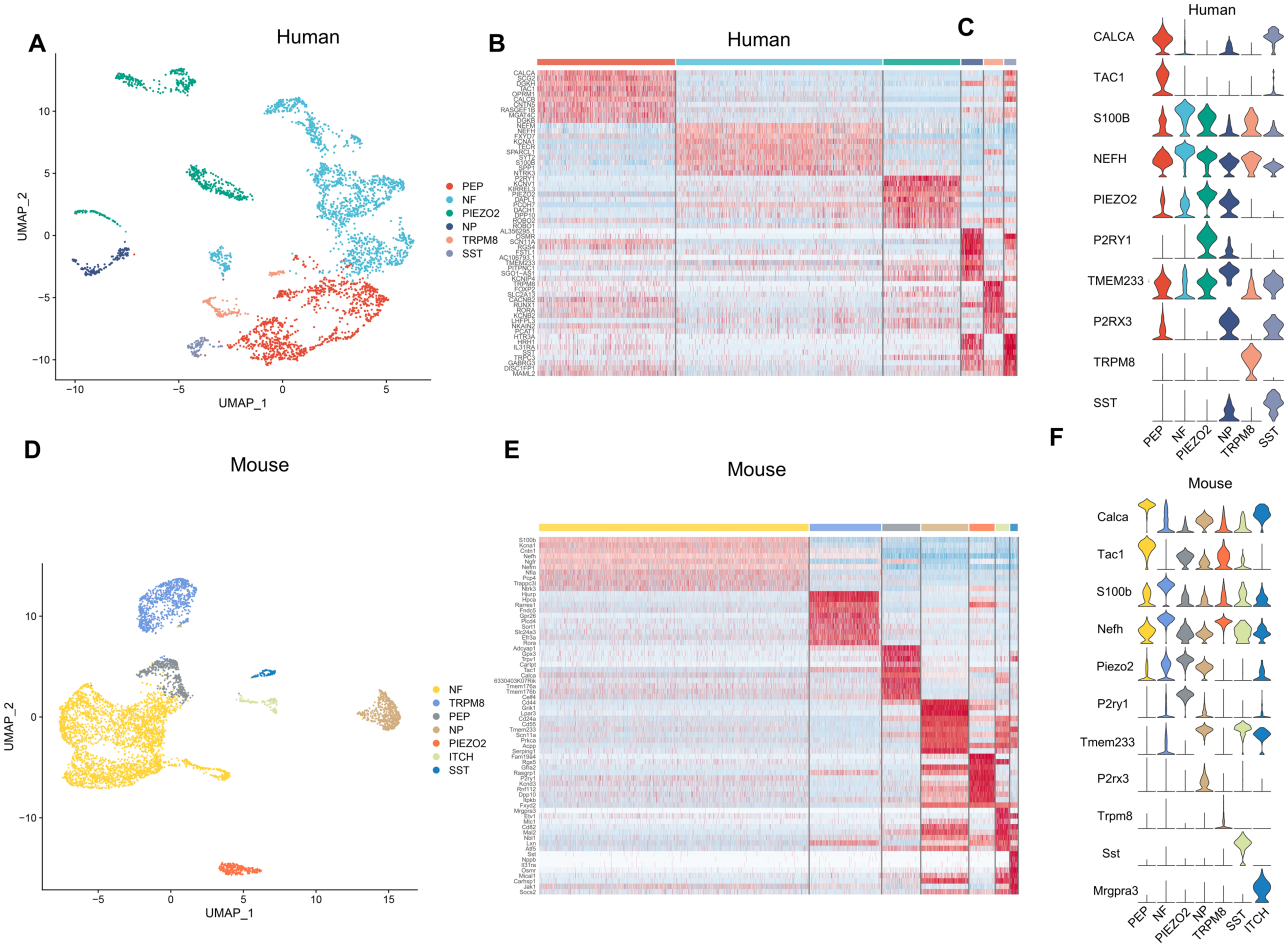
Analysis of neuron subtypes in the TG. **(A)** The UMAP plot of neuron subtypes in the human TG. **(B)** The gene heat map of the top 10 genes for neuron subtypes in the human TG. **(C)** The violin plot indicating the signature marker genes of the neuron subtypes in the human TG. **(D)** UMAP plot of neuron subtypes in the mouse TG. **(E)** The gene heat map of top 10 genes for neuron subtypes in the mouse TG. **(F)** The violin plot indicating the signature marker genes of the neuron subtypes in the mouse TG.

To further investigate the transcriptional patterns of human and mouse neurons within the TG, we extracted the neurons and then performed further clustering to classify the neurons into different subtypes. Marker genes and annotations of neuronal subtypes were referred to other published scRNA-seq or snRNA-seq studies on sensory ganglia (Liu et al., 2022; Yang et al., 2022). The human neurons of TG consisted of six subtypes (Figure 5A). The gene heatmap showed the top 10 marker genes for the six neuron subtypes of humans (Figure 5B). The neuron subtypes in the human TG and the markers used were also listed: PEP (peptidergic nociceptors) (*CALCA*, *TAC1*), NP (non-peptidergic nociceptors) (*P2RX3*, *TMEM233*), NF neurons (myelinated neurons, high expression of NEFH neurons) (*NEFH*, *S100B*), PIEZO2 neurons (high expression of *PIEZO2* neurons) (*PIEZO2*, *P2RY1*), TRPM8 neurons (high expression of *TRPM8* neurons) (*TRPM8*), and SST neurons (high expression of *SST* (Somatostatin) neurons) (*SST*) (Figure 5C).

The mouse neurons in the TG consisted of seven subtypes (Figure 5D). The top 10 marker genes for the seven neuron subtypes of the mouse TG were demonstrated in the gene heatmap (Figure 5E). The mouse neurons in the TG consisted of seven subtypes including PEP neurons (*Calca*, *Tac1*), NP neurons (*P2rx3*, *Tmem233*), NF neurons (*Nefh*, *S100b*), Piezo2 neurons (*Piezo2*, *P2ry1*), Trpm8 neurons (*Trpm8*), Sst neurons (*Sst*), ITCH neurons (high expression of *Mrgpra3* neurons) (*Mgpra3*) (Figure 5F).

### The expression profile of pain-related genes in neuron subtypes

**Figure 6:**
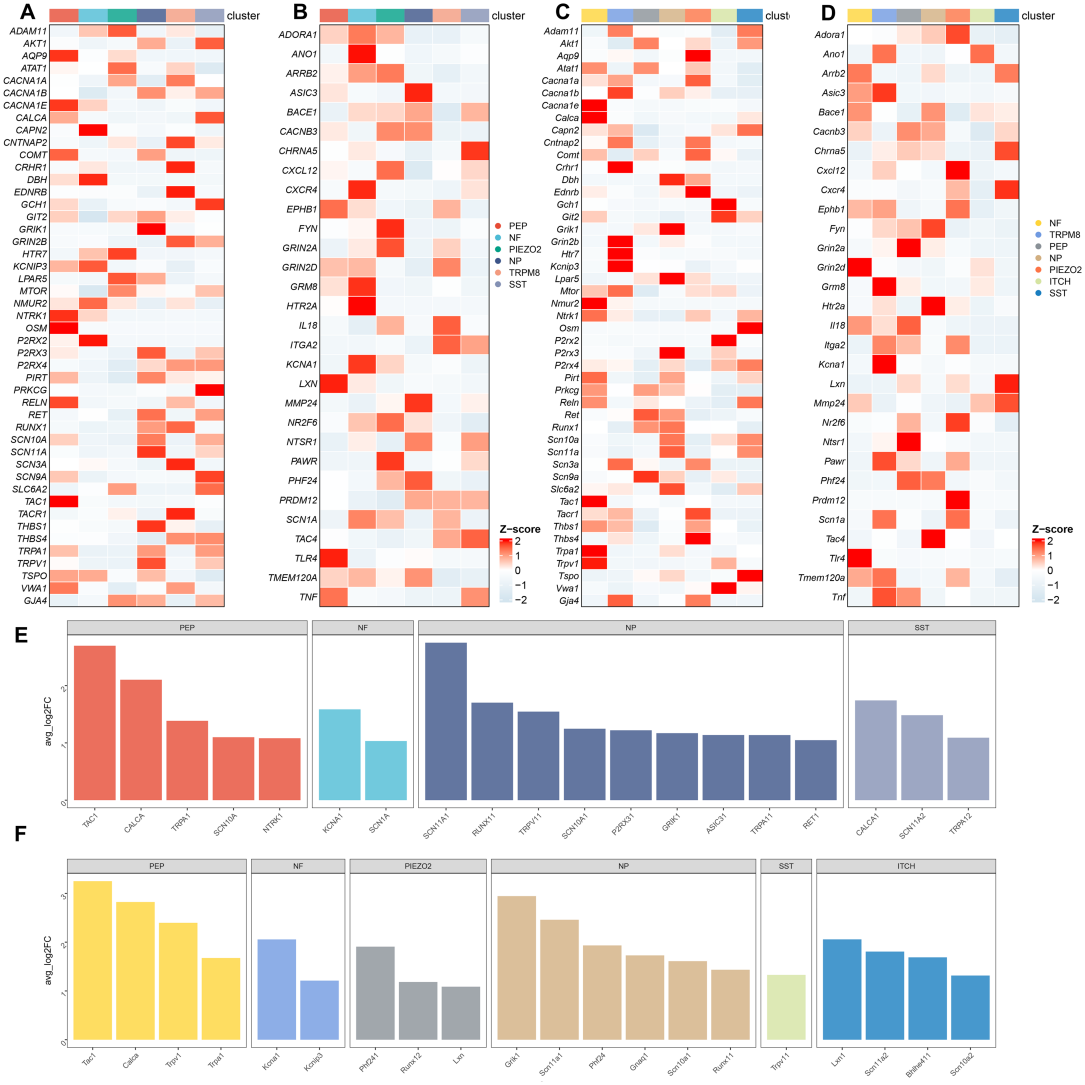
Transcriptional landscape of pain-related genes in neuron subtypes of the TG. **(A)** The gene heatmap showing the expression of genes associated with “response to pain” in neuron subtypes of the human TG. **(B)** The gene heatmap showing the expression of genes associated with “detection of stimulus involved in sensory perception of pain” in neuron subtypes of the human TG. **(C)** The gene heatmap showing the expression of genes associated with “response to pain” in neuron subtypes of the mouse TG. **(D)** The gene heatmap showing the expression of genes associated with “detection of stimulus involved in sensory perception of pain” in neuron subtypes of the mouse TG. **(E,F)** The bar plots indicating significant differences in the expression of pain-related genes among neuron subtypes of the human **(E)** and the mouse **(F)** TG. The horizontal axis of the bars represents neuron subtypes distinguished by different colors, and the vertical axis of the bars represents log2-fold change (log2FC). Genes with log2FC > 1 and a Wilcoxon Rank Sum test *p* < 0.01 are shown.

We further investigated the expression and distribution of pain-related genes in the human and mouse neuronal subtypes. The expression of genes associated with “response to pain” in neuron subtypes in human and mouse TG was shown in Figures 6A,B, respectively. The expression of genes associated with “detection of stimulus involved in sensory perception of pain” in the neuron subtypes in human and mouse TG was shown in Figures 6C,D, respectively. To compare the differences in the expression of pain-related genes in different neuronal subtypes, we performed the differential gene expression analysis among distinct neuron subtypes of human and mouse TG using the FindMarkers function. Genes with a *p*-value less than 0.01 and log2 fold change more than 1 were considered significantly differentially expressed. The differentially expressed genes of the neuron subtypes in human and mouse TG are shown in the bar plots (Figures 6E,F).

### The expression profile of circadian rhythms-related genes in neuron subtypes

The distribution of the core clock genes in the neuronal subtypes of the TG did not show significant inter-subtype difference, as shown in Supplementary Figures S1A,B. The expression of melatonin-related genes in the neuron subtypes of human and mouse TG is illustrated in Supplementary Figures S1C,D, respectively. In human TG, *NOS1* was more enriched in TRPM8 and SST neurons, and *NOS2* was enriched in PEP and SST neurons, which was hard to detect in mouse neurons. *RORA* was most abundantly expressed within human and mouse TRPM8 neurons, relative to other neuron subtypes. As shown in Supplementary Figure S1D, compared to human data, *Cand2* is expressed in a very low percentage in mouse PEP neurons. The expression of opioid-related genes in the neuron subtypes of human and mouse TG is demonstrated in Supplementary Figures S1E,F, respectively. In the opioid receptor genes, *OPRD1*, which encodes opioid receptor delta 1, was enriched in all human neuron subtypes except PIEZO2 neurons. *OPRK1* (Opioid Receptor Kappa 1) was only clearly found in NP and SST neurons in human TG. In addition, *OPRM1* (Opioid Receptor Mu 1) was more abundant in PEP (70.3%) and SST (87.1%) neurons of human TG, which was significantly higher than the proportion of PEP (5.0%) and SST (5.3%) in mouse TG.

## Discussion

The TG plays a vital role in the processing and transmitting orofacial sensory information. Research has shown that various cell types within the TG, including neurons, SGCs, and macrophages, are involved in pain production, transmission, and maintenance (McDonald et al., 2014; Ji et al., 2016; Liu et al., 2022). Although a few studies have explored the role of the DRG in regulating somatic pain rhythms (Zhang et al., 2012), investigations into the involvement of the TG in orofacial pain rhythms are limited. To address this gap, in this study, we used snRNA-seq data from human and mouse TG to construct a comprehensive cellular atlas of the TG in the physiological state. Furthermore, we generated expression profiles of core clock genes, pain-related genes, as well as melatonin and opioid-related genes in the TG. We further classified the neurons of the TG to obtain different neuron subtypes and demonstrated the expression and localization of these genes in neuron subtypes of the TG. Additionally, we compared the transcriptional profiles of these genes in the TG between human and mouse samples to identify species-specific differences. This study, to our knowledge, is the first to reveal cell-type specific molecular signatures of the circadian clock and pain-related genes in mouse and human TG. Our results provide a valuable preliminary resource for studying the molecular mechanisms of orofacial pain rhythms.

Relative to traditional bulk RNA sequencing, scRNA-seq and snRNA-seq offer opportunities for the discovery of novel biological insights, including the identification of cell types, states, and lineage trajectories, as well as the ability to probe cellular heterogeneity at the transcriptional level (Tanay and Regev, 2017). Unlike scRNA-seq, snRNA-seq analyzes single nuclei instead of single cells, which solves the problem of isolating neural tissues and other tissues into single-cell suspensions and frozen samples for sequencing, facilitating human tissue sequencing, while minimizing cellular transcriptional changes caused by dissociation (Lacar et al., 2016). In the snRNA-seq analysis of this study, we reveal that the human and mouse TG have the same cell types, including six major cell clusters, which are consistent with other published scRNA-seq studies on sensory ganglia (Avraham et al., 2020; Mapps et al., 2022; Chu et al., 2023). Moreover, traditional approach neurons are classified according to anatomical, physiological, and biochemical characteristics, but the heterogeneity of neurons in sensory ganglia at the transcriptional level is not well understood. The classification of neuronal subtypes was referred to the published literature (Yang et al., 2022). But we used the highly expressed gene *Piezo2* to name this class of neuronal subtypes in human and mouse TG. We identified a new class of neuron subtypes that significantly overexpressed Mgpra3 in mouse TG and named them ITCH neurons. The presence of *Mgrpa3* as a marker for ITCH neurons has also been present in other scRNA-seq studies (Li et al., 2016) and is actually a non-peptidergic neuron in DRG (Parpaite et al., 2021).

Animals have evolved internal timing systems called biological clocks to adapt and actively respond to the light/dark cycles of the environment. It is widely believed that the central and peripheral circadian clocks co-regulate circadian rhythms in mammals. The central circadian clock is located in the optic suprachiasmatic nucleus (SCN) (Dibner et al., 2010), and the SCN neurons synchronize the coupled generated circadian rhythms to peripheral organs and tissues (Mohawk et al., 2012). Apart from the SCN, peripheral organs and tissues also have independent peripheral circadian clocks. In the liver, pancreas, lungs, and muscles, the peripheral clock is involved in regulating physiological processes, including glucose metabolism, immune response, and myogenesis (Sherratt et al., 2019). In this study, we revealed the expression landscape of core clock genes in different cell types of TG, including neurons, SGCs, SCs, ICs, ECs, and fibroblasts. The core clock genes were abundantly expressed in different types of cell clusters of TG, which is consistent with previous studies demonstrating that clock genes are expressed in most peripheral tissues (Franken and Dijk, 2009). The results of this study indicated that *RORA* was the most abundantly expressed clock gene in both human and mouse TG. Previous research has indicated the relationship between *RORA* and pain conditions. For instance, it has been suggested that MicroRNA-19b may be involved in post-traumatic widespread pain (PTWP) and post-traumatic stress symptoms (PTSS) through the regulation of *RORA* (Linnstaedt et al., 2020). In addition, RORα has also been identified as a lamina-specific transcription factor in the dorsal horn of the spinal cord (Li et al., 2006). In our results, *ARNTL* (or *BMAL1*) was also enriched in the human and mouse TG. A study found that *BMAL1:CLOCK* modulates inflammatory rhythmic pain by regulating the oscillatory expression of substance P (SP) in the dorsal root ganglion (DRG) (Zhang et al., 2012). Furthermore, *PER1* was commonly expressed in various cell clusters and neuron subtypes in human and mouse TG in our results (Figures 2A,B). In a neuropathic pain model, the expression of *Per1* mRNA and protein was significantly suppressed in the spinal dorsal horn of mice after partial sciatic nerve ligation, which may contribute to the induction of neuropathic pain (Morioka et al., 2016).

In this study, species differences were observed in the expression of core clock genes in human and mouse TG, such as *CRY1*, *PER2*, and *NR1D1*. In fact, there are differences in the sleep–wake cycles of humans and mice, and the rhythmic oscillations of the clock genes are not identical (Yan et al., 2008). Therefore, this may account for the species differences in the cellular distribution of clock genes in peripheral tissues, including TG.

Our results showed that *PIRT* was specifically enriched in neurons of both the human and mouse TG. PIRT (Phosphoinositide-Interacting Regulator of TRPV1) is a membrane protein expressed in neurons of the DRG (Gao et al., 2021) that modulates neuropathic pain by binding to TRPV1 to enhance its activity and promote the expression of TRPV1 channels (Wang et al., 2018). TRPV1 was enriched in neurons of the human and mouse TG (Figures 3A,B), and was expressed at higher levels in human PEP and SST neuron subtypes (Figure 6E), but at a higher level in mouse NP neuron subtypes (Figure 6F). TRPV1 is an ion channel sensitive to capsaicin and injurious stimuli, causing a burning sensation, and is suggested to play a role in a variety of chronic pains (Iftinca et al., 2021). Moreover, the diurnal expression of TRP channels was also observed in the DRG in chemotherapy-induced neuropathic pain (Kim et al., 2020). *RET* (Ret Proto-Oncogene) was also specifically enriched in neurons of both the human and mouse TG (Figures 3A,B). In an animal model of bone cancer pain, RET can bind to the ligand GDNF to activate the downstream ERK signaling pathway, and promote sensitization of DRG neurons (Yuan et al., 2022). *EDNRB* (Endothelin Receptor Type B) is one of the genes abundantly expressed in the SGCs of both the human and mouse TG (Figures 3A,B). It has been shown that EDNRB is upregulated in astrocytes of the spinal cord and that its antagonist BQ788 inhibits glial cell activation and pain symptoms (Yamasaki et al., 2016). However, no studies have been reported on the role of EDNRB in sensory ganglion SGCs in pain. Our results demonstrated that *COMT* (Catechol-O-Methyltransferase) was enriched in both human and mouse SGCs in the TG (Figures 3A,B). In addition, a study has revealed that COMT was a downstream regulatory target of NF-κB in inflammatory pain models (Hartung et al., 2015). *ASIC3* (Acid Sensing Ion Channel Subunit 3) was specifically expressed in neurons of the human and mouse TG, and was more abundantly expressed in NP neurons than in other subtypes for the human TG (Figure 6E). ASIC3 is a pH sensor that responds to slight extracellular acidification (Qian et al., 2021), and was found obviously enhanced with TRPV1 in the DRG in rats of bone cancer models (Wu et al., 2012).

Melatonin exerts its effect on cell function by interacting with various target proteins, including receptors, enzymes, and transporters. Our snRNA-seq data demonstrated that *NOS1* was expressed specifically in neurons of human TG (Figure 4A). Furthermore, melatonin can alleviate neuropathic pain by inhibiting neuronal NOS expression in DRG neurons (Lin et al., 2017). NO/NOS is also very important for the circadian clock system and NO is involved in the transmission of diurnal light information from the eye to the SCN (Golombek et al., 2004). Opioid receptors, such as mu, delta, and kappa opioid receptors, are distributed in various regions of the brain that are involved in the regulation of circadian rhythms. Changes in MOR mRNA expression in the periaqueductal gray synchronize with diurnal changes in pain thresholds for thermal stimulation (Takada et al., 2013). The re-expression of OPRM1 in a cancer pain model may produce antinociception by mediating the effect of endogenous opioids (Viet et al., 2014), and OPRM1 was also one of the genes with rhythmic oscillations in neuropathic pain (Kim et al., 2020). Our results illustrated the rich expression of *Oprm1* in human TG neurons.

In this study, we constructed the transcriptional landscape of rhythm-related and pain-related genes in cell clusters and neuron subtypes in human and mouse TG. However, we did not perform scRNA-seq directly through the TG of mice with rhythmic pain at different time points to screen for potential regulatory genes that may play a role in orofacial pain. This is a direction we need to explore in the future. We expect that our results, by constructing the atlas of circadian clock genes, pain-related genes, melatonin, and opioid-related genes in cells of TG, can provide a preliminary resource for studying rhythmic pain in the orofacial region.

## Data availability statement

The datasets presented in this study can be found in online repositories. The names of the repository/repositories and accession number(s) can be found at: https://www.ncbi.nlm.nih.gov/geo/, GSE 197289.

## Ethics statement

The patients/participants provided their written informed consent to participate in this study.

## Author contributions

YC: conceptualization, methodology, formal analysis, and original draft writing. YW: software and methodology. SJ: methodology and formal analysis. KX: original draft writing. JL: review and editing. LM: methodology. WF: conceptualization and review and editing. FH: supervision, methodology, and funding acquisition. All authors contributed to the article and approved the submitted version.

## Funding

This work was supported by the National Natural Science Foundation of China (Grant Nos. 81870737, 81771098, and 82270997), Natural Science Foundation of Guangdong Province (Grant No. 2021A1515011779) and Guangdong Financial Fund for High-Caliber Hospital Construction (Grant No. 174-2018-XMZC-0001-03-0125/D-02).

## Conflict of interest

The authors declare that the research was conducted in the absence of any commercial or financial relationships that could be construed as a potential conflict of interest.

## Publisher’s note

All claims expressed in this article are solely those of the authors and do not necessarily represent those of their affiliated organizations, or those of the publisher, the editors and the reviewers. Any product that may be evaluated in this article, or claim that may be made by its manufacturer, is not guaranteed or endorsed by the publisher.
