# Supplementary figures and images for: Single-nucleus transcriptome analysis reveals transcriptional profiles of circadian clock and pain related genes in human and mouse trigeminal ganglion
Source: Front Neurosci. 2023 May 11;17:1176654. doi: 10.3389/fnins.2023.1176654 (PMC10210144; doi:10.3389/fnins.2023.1176654)

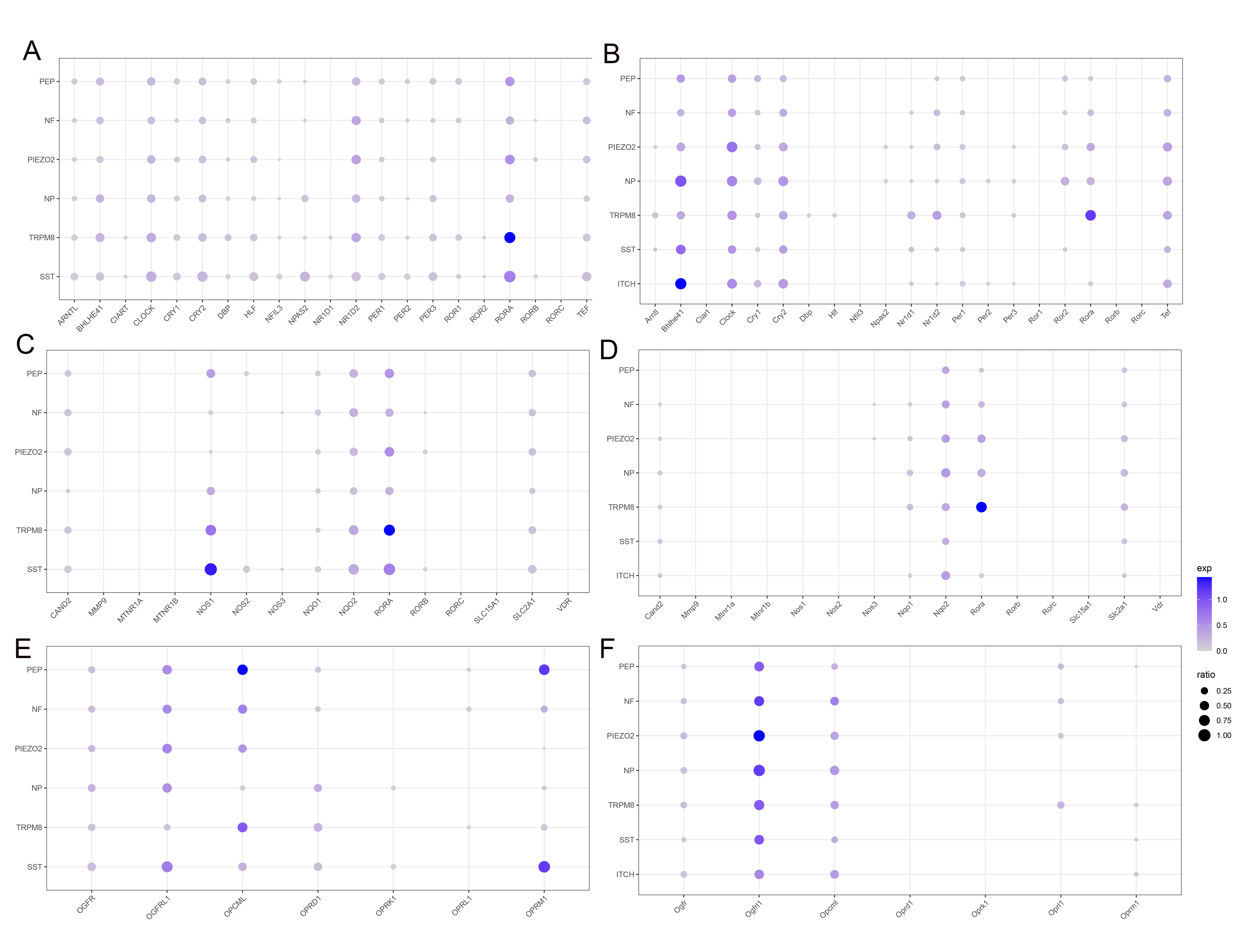

Supplement: Supplementary file 1 [file Image_1.TIF]
